# Supplementary material for: Controlling the sign of optical forces using metaoptics
Source: Nat Commun. 2026 Jan 9;17:1163. doi: 10.1038/s41467-025-67928-6 (PMC12858989; doi:10.1038/s41467-025-67928-6)
Supplement: Supplementary file 1 — Supplementary Information [file 41467_2025_67928_MOESM1_ESM.pdf]

Supplementary Information: Controlling the sign of optical forces using metaoptics

Adeel Afridi,[1,2] Bruno Melo,[1,2] Nadine Meyer,[1,2,*] and Romain Quidant[1,2]

[1] *Nanophotonic Systems Laboratory, Department of Mechanical and Process Engineering, ETH Zurich, 8092 Zurich, Switzerland*

[2] *Quantum Center, ETH Zurich, 8083 Zurich, Switzerland*

(Dated: December 10, 2025)

This Supplementary Information provides additional theoretical, numerical, and experimental details supporting the results presented in the main text. In Sec. I of this supplementary material we describe the experimental setup, the generation of the time dependent optical driving force $F(t)$ and the displacement detection. In Sec. II, we present the multipole decomposition of the optical gradient force in a standing wave. In Sec. III, we provide details on the finite-element simulations performed in COMSOL, followed by a discussion of the effective polarizability and its force contributions from higher-order Mie resonances. The corresponding electric and magnetic near-field distributions along with the far-field scattering are also presented. Finally, we examine the polarization independence of the optical force, the emergence of optical torque under circularly polarized excitation, and the force response in the visible spectral range. In Sec. IV we study the model of a driven, underdamped harmonic oscillator and give the analytical expressions for its amplitude and phase response. The last two sections study the motion of a flat mirror membrane used for normalization (Sec. V), and the comparison of the motion of a structured and a mirror membrane under thermal and optical driving (Sec. VI).

I. EXPERIMENTAL SETUP

A complete layout of the experimental setup is shown in Fig. S1. We use a laser source at $\lambda = 1550\,\mathrm{nm}$ to drive and probe the membrane's motion. The laser beam is split into two components: the p-polarized light serves as the probe beam, while the s-polarized light generates a standing wave and drives the membrane at the driving frequency ω_{dr} (pump beam). The membrane's motion is detected via homodyne measurement of the back-reflected probe beam using a lock-in amplifier. The details of the optical setup are described below.

A. Amplitude-Modulated Standing-Wave Pump Beam

The s-polarized pump beam (E_s) is amplitude-modulated using an acousto-optic modulator (AOM), generating the zeroth-order ($E_{s,0}$) and first-order ($E_{s,+1}$) diffraction components. These beams are then expanded and separated from each other using a pair of lenses ($f_1 = 100$ mm, $f_2 = 400$ mm).

Next, $E_{s,0}$ and $E_{s,+1}$ are split into two beams via a 50:50 beam splitter (BS3). One set of beams ($E_{s,0}$ and $E_{s,+1}$) is directed onto the membrane from the left via a polarizing beam splitter (PBS3) and an objective lens (Obj1, NA = 0.4, 20×). The other set propagates through another lens system ($f_3 = f_4 = 200$ mm) and is routed to a piezo-actuated mirror (PM2) via PBS4. This mirror allows precise control over the optical path length, thereby adjusting the phase $\Delta\phi$ of the incoming light. The reflected beams ($E_{s,0}^{\Delta\phi}$ and $E_{s,+1}^{\Delta\phi}$) are then focused onto the membrane using a second objective lens (Obj2, NA = 0.4, 20×).

* nmeyer@ethz.ch

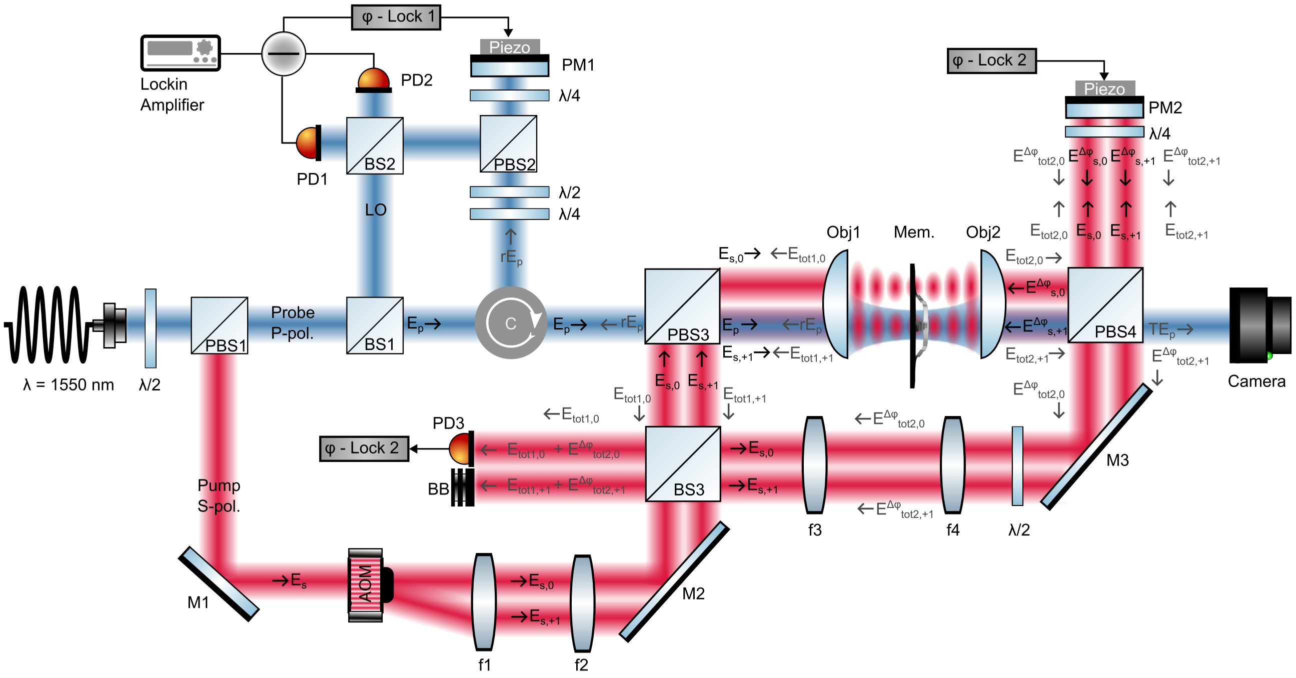

Figure S1. **Experimental setup for driving and detecting membrane motion.** A laser source at $\lambda = 1550\,\mathrm{nm}$ is used to drive and probe the mechanical motion of the membrane. The beam is split into two polarization components: s-polarized light (red) serves as the pump and is amplitude-modulated via an acousto-optic modulator (AOM) to generate zeroth- and first-order diffraction components, which are split and directed from opposite sides to form a standing wave at the membrane. A piezo-actuated mirror (PM2) controls the phase difference $\Delta\phi$ between counter-propagating pump beams. The p-polarized probe beam (blue) is also split, with one part acting as the local oscillator (LO) and the other probing the membrane through a circulator. The back-reflected probe interferes with the LO on a balanced photodetector, and the resulting homodyne signal is processed using a lock-in amplifier to extract the membrane's driven motion.

These two counter-propagating sets of pump beams form a standing wave, one for each diffraction order. The zeroth-order standing wave interacts with the unstructured (flat) region of the membrane, while the first-order standing wave interacts with the metasurface. The relative position of the standing wave nodes and antinodes with respect to the membrane can be fine-tuned by adjusting PM2.

To lock and stabilize the phase $\Delta\phi$ against slow drifts, we monitor the signals $E_{tot1,0}$ and $E^{\Delta\phi}_{tot2,0}$ using a photodetector (PD3). The output from PD3 is fed into a proportional controller (ϕ-Lock 2), which actively stabilizes PM2. These signals are defined as follows:

$$E_{tot1,0} = T_0 E^{\Delta\phi}_{s,0} + r_0 E_{s,0} \tag{1}$$

$$E^{\Delta\phi}_{tot2,0} = T_0 E^{\Delta\phi}_{s,0} + r_0 E^{2\Delta\phi}_{s,0} \tag{2}$$

where T_0 and r_0 represent the transmission and reflection coefficients of the unstructured membrane region, respectively.

B. Probe Beam and Homodyne Detection

The p-polarized probe beam is split by a beam splitter (BS1) into two components: one serving as the local oscillator (LO) and the other (E_p) passing through a circulator (C). The probe beam is then combined with the first-order pump beam ($E_{s,+1}$) in PBS3 and is focused onto the metasurface region of the membrane by Obj1.

The back-reflected probe beam (rE_p) is extracted through the reflection port of the circulator and directed toward a piezo-actuated mirror (PM1), which fine-tunes the optical path. The reflected probe is then mixed with the LO using BS2 and detected by a balanced photodetector (PD1, PD2). The homodyne phase is actively stabilized by a proportional controller (ϕ-Lock 1), which drives PM1.

Finally, the stabilized homodyne signal is fed into a lock-in amplifier to extract the amplitude and phase of the membrane's motion.

II. MULTIPOLE DECOMPOSITION OF THE GRADIENT FORCE IN A STANDING WAVE

We assume two weakly focused, linear polarized, counterpropagating Gaussian beams along z with relative phase $\phi = 0$ in vacuum. Each beam has an on-axis electric amplitude E_0 and magnetic amplitude H_0.

$$\mathbf{E}(\mathbf{r}) = \hat{\mathbf{x}}\, 2E_0\, e^{-r^2/w^2} \cos(kz), \qquad r^2 = x^2 + y^2 \tag{3}$$

$$\mathbf{H}(\mathbf{r}) = \hat{\mathbf{y}}\, 2H_0\, e^{-r^2/w^2} \sin(kz) \tag{4}$$

$$|\mathbf{E}|^2 = 4E_0^2\, e^{-2r^2/w^2} \cos^2(kz) \tag{5}$$

$$|\mathbf{H}|^2 = 4H_0^2\, e^{-2r^2/w^2} \sin^2(kz) \tag{6}$$

so that the intensity nodes/antinodes of $\mathbf{H}$ are shifted by $\lambda/4$ relative to $\mathbf{E}$. Following [1], we write the total conservative (gradient) force as the individual contributions of each multipole [2]

$$\langle \mathbf{F} \rangle \approx \langle \mathbf{F}_{ed} \rangle + \langle \mathbf{F}_{md} \rangle + \langle \mathbf{F}_{eq} \rangle + \langle \mathbf{F}_{mq} \rangle + \langle \mathbf{F}_{eo} \rangle + \langle \mathbf{F}_{mo} \rangle \tag{7}$$

where $\langle \mathbf{F}_{ed} \rangle$ is the time averaged gradient force due to the electric dipole, $\langle \mathbf{F}_{md} \rangle$ the magnetic dipole, $\langle \mathbf{F}_{eq} \rangle$ the electric quadrupole, $\langle \mathbf{F}_{mq} \rangle$ the magnetic quadrupole, $\langle \mathbf{F}_{eo} \rangle$ the electric octupole and $\langle \mathbf{F}_{mo} \rangle$ the magnetic octupole with

$$\langle \mathbf{F}_{ed} \rangle \propto \Re[\nabla(\mathbf{p} \cdot \mathbf{E}^*)] \tag{8}$$

$$\langle \mathbf{F}_{md} \rangle \propto \Re[\nabla(\mathbf{m} \cdot \mathbf{H}^*)] \tag{9}$$

$$\langle \mathbf{F}_{eq} \rangle \propto \Re[\nabla(\mathbf{Q_e} : \nabla\mathbf{E}^*)]$$

$$\langle \mathbf{F}_{mq} \rangle \propto \Re[\nabla(\mathbf{Q_m} : \nabla\mathbf{H}^*)] \tag{10}$$

$$\langle \mathbf{F}_{eo} \rangle \propto \Re[\nabla(\mathbf{O_e} : \nabla\nabla\mathbf{E}^*)]$$

$$\langle \mathbf{F}_{mo} \rangle \propto \Re[\nabla(\mathbf{O_m} : \nabla\nabla\mathbf{H}^*)] \tag{11}$$

$$\tag{12}$$

Hereby, ·" represents the single contraction and ":" the double contraction used as

$$[\mathbf{Q_e} : \nabla\mathbf{E}^*]_{ij} = Q_{ij}^{(e)} \partial_i E_j^*$$

$$[\mathbf{O_e} : \nabla\nabla\mathbf{E}^*]_{ijk} = O_{ijk}^{(e)} \partial_i \partial_j E_k^*$$

The mulitpole moments are given by

$$p_i = \epsilon_0 \alpha_d^{(e)} E_i \qquad \text{electric dipole moment} \tag{13}$$

$$m_j = \alpha_d^{(m)} H_i \qquad \text{magnetic dipole moment} \tag{14}$$

$$Q_{ij}^{(e)} = \epsilon_0 \alpha_q^{(e)} \mathcal{S}[\partial_i E_j] \qquad \text{electric quadrupole moment} \tag{15}$$

$$Q_{ij}^{(m)} = \alpha_q^{(m)} \mathcal{S}[\partial_i H_j] \qquad \text{magnetic quadrupole moment} \tag{16}$$

$$O_{ijk}^{(e)} = \varepsilon_0\, \alpha_o^{(e)} \mathcal{S}\big[\partial_i \partial_j E_k\big] \qquad \text{electrical octupole moment} \tag{17}$$

$$O_{ijk}^{(m)} = \alpha_o^{(m)} \mathcal{S}\big[\partial_i \partial_j H_k\big] \qquad \text{magnetic octupole moment} \tag{18}$$

where $\mathcal{S}[\partial_i E_j] = \frac{1}{2}(\partial_i E_j + \partial_j E_i) + \frac{1}{3}\delta_{ij}(\nabla\mathbf{E})$ and $\mathcal{S}[\partial_i \partial_j E_k] = \frac{1}{3}\left(\partial_i \partial_j E_k + \partial_i \partial_k E_j + \partial_k \partial_j E_i\right) - \frac{1}{5}(\delta_{ij} u_k + \delta_{ik} u_j + \delta_{jk} u_i)$ with $u_l = \frac{1}{3}(\nabla^2 E_l + 2\partial_l(\nabla \cdot \mathbf{E}))$. For a homogeneous sphere the spherical-multipole/Mie coefficients a_l, b_l (electric, magnetic) map to the usual dimensionful polarizabilities as follows.

$$\alpha_l^{(e)} = i\frac{(2l+1)(l+1)}{l}\frac{\pi}{k^{2l+1}}a_l \tag{19}$$

$$\alpha_l^{(m)} = i\frac{(2l+1)(l+1)}{l}\frac{\pi}{k^{2l+1}}b_l \tag{20}$$

where $\alpha^{(e),(m)}$ are the multipole polarisabilites ($l = 1$ dipole d, $l = 2$ quadrupole q, $l = 3$ octupole o).

In the following we will illustrate that for the special case of a linearly polarized standing wave the gradient force due to different multipoles scale with the intensity gradient such that we can assign an approximate effective polarisablity

$$\alpha_{\text{eff}} \approx [\alpha_d^{(m)} - \alpha_d^{(e)}] - [\alpha_q^{(m)} - \alpha_q^{(e)}] + [\alpha_o^{(m)} - \alpha_o^{(e)}].$$

To do so, we will first show that the sign alternates between different electrical orders (dipole, quadrupole and optupole) and then we use the example of the magnetic and electric dipole to show that the same order multipole differs in sign between electrical and magnetic multipoles. We assume a simplified case of on axis forces F_z with $x = y = 0$.

A. Electrical multipoles

a. Electrical dipole: With the electric dipole polarizability $\alpha_d^{(e)}$ in $\mathbf{p} = \varepsilon_0\alpha_d^{(e)}\mathbf{E}$, the time-averaged conservative part due to the electric dipole is

$$\langle \mathbf{F}_{ed}\rangle \propto \Re[\nabla(\mathbf{p}\cdot\mathbf{E}^*)] \propto \Re\{\alpha_d^{(e)}\}\,\nabla|\mathbf{E}|^2. \tag{21}$$

Thus, on axis ($x = y = 0$) we get a restoring force toward the intensity maxima if $\Re\{\alpha_d^{(e)}\} > 0$:

$$F_{ed,z}(0,0,z) \propto -\Re\{\alpha_d^{(e)}\}\,k\,E_0^2\,\sin(2kz). \tag{22}$$

b. Electrical quadrupole: The gradient force due to the electrical quadrupole s given by

$$\langle \mathbf{F}_{eq}\rangle \propto \Re[\nabla(\mathbf{Q_e} : \nabla\mathbf{E}^*)] \tag{23}$$

where $\mathbf{Q_e} : \nabla\mathbf{E}^* = \Sigma_{i,j}Q_{ij}^{(e)}(\partial_j E_i^*)$ with $Q_{ij}^{(e)} = \epsilon_0\alpha_q^{(e)}\mathcal{S}[\partial_i E_j]$ and $\alpha_q^{(e)}$ electric quadrupole polarizability. Evaluation of the force on the optical axis ($x = y = 0$) and because only $E_x \neq 0$, the only nonzero components are

$$[\mathbf{Q_e} : \nabla\mathbf{E}^*]_{zx} = \varepsilon_0\alpha_q^{(e)}\mathcal{S}[\partial_z E_x]\partial_z E_x^* = 2\varepsilon_0\alpha_q^{(e)}k^2E_0^2\sin^2(kz)$$

with $\partial_z E_x = -kE_0\sin(kz)$ and $\mathcal{S}[\partial_z E_x] = \partial_z E_x/2$. This yields

$$F_{eq,z}(0,0,z) \propto \Re\left\{\alpha_q^{(e)}\right\}k^2E_0^2\partial_z\sin^2(kz) \propto \Re\left\{\alpha_q^{(e)}\right\}k^3E_0^2\sin(2kz) \tag{24}$$

c. Electric octupole: The gradient force due to the electrical octupole is

$$\left\langle \mathbf{F}_{eo}\right\rangle \propto \Re\{\nabla(\mathbf{O_e} : \nabla\nabla\mathbf{E}^*)\} \tag{25}$$

where $\mathbf{O_e}$ be $O_{ijk}^{(e)}$ is the electric octupole induced by the local second derivatives of the field,

$$O_{ijk}^{(e)} = \varepsilon_0\,\alpha_o^{(e)}\,\mathcal{S}\big[\partial_i\partial_j E_k\big], \tag{26}$$

and $\alpha_o^{(e)}$ is the electric octupole polarizability. For the contraction

$$[\mathbf{O_e} : \nabla\nabla\mathbf{E}^*]_{ijk} = \varepsilon\,\alpha_o^{(e)}\mathcal{S}[\partial_i\partial_j E_k]\partial_i\partial_j E_k^*$$

we only need to consider three terms due to the fact that

$$\partial_i\partial_j E_k = 0\;\forall k = y,z \quad \text{since} \quad \mathbf{E} = (E_x,0,0)$$

and

$$\partial_i \partial_j E_k = 0 \, \forall i \neq j \quad \text{for} \quad x = y = 0.$$

The remaining terms are

$$\mathcal{S}_{xxx}\partial_x^2 E_x^* = (\partial_x^2 E_x - \frac{1}{5}(\nabla^2 E_x + 2\partial_x^2 E_x))\partial_x^2 E_x^* \approx -\frac{2k^2}{5w^2}|E_x|^2 \tag{27}$$

$$\mathcal{S}_{yyx}\partial_y^2 E_x^* = (\frac{1}{3}\partial_y^2 E_x - \frac{1}{15}(\nabla^2 E_x + 2\partial_x^2 E_x))\partial_y^2 E_x^* \approx -\frac{2k^2}{15w^2}|E_x|^2 \tag{28}$$

$$\mathcal{S}_{zzx}\partial_z^2 E_x^* = (\frac{1}{3}\partial_z^2 E_x - \frac{1}{15}(\nabla^2 E_x + 2\partial_x^2 E_x))\partial_z^2 E_x^* \approx \frac{4}{15}k^4|E_x|^2 \tag{29}$$

where we assumed $x = y = 0$ and the paraxial approximation $kw \gg 1$.

$$[\mathbf{O_e} : \nabla\nabla\mathbf{E}^*] = \varepsilon\, \alpha_o^{(e)} \left(\mathcal{S}_{xxx}\partial_x^2 E_x^* + \mathcal{S}_{yyx}\partial_y^2 E_x^* + \mathcal{S}_{zzx}\partial_z^2 E_x^*\right) \tag{30}$$

$$\approx \frac{4}{15}\varepsilon\, \alpha_o^{(e)} k^4 |E_x|^2 \tag{31}$$

$$F_{eo,z}(0,0,z) \propto \Re\left\{\alpha_o^{(e)}\right\} k^4 \partial_z[4E_0^2\cos^2(kz)] = -\Re\{\alpha_O^{(e)}\}\, k^5 E_0^2\, \sin(2kz) \tag{32}$$

d. Comparison of on axis forces due to electrical dipole, quadrupole, octupole contributions From the earlier results for the same field, we get

$$F_{ed,z}(0,0,z) \propto -\Re\{\alpha_d^{(e)}\}\, k\, E_0^2\, \sin(2kz) \tag{33}$$

$$F_{eq,z}(0,0,z) \propto +\Re\{\alpha_q^{(e)}\}\, k^3 E_0^2\, \sin(2kz) \tag{34}$$

$$F_{eo,z}(0,0,z) \propto -\Re\{\alpha_o^{(e)}\}\, k^5 E_0^2\, \sin(2kz). \tag{35}$$

Similar considerations for the magnetic multipoles yield

$$F_{md,z}(0,0,z) \propto \Re\{\alpha_d^{(m)}\}\, k\, H_0^2\, \sin(2kz) \tag{36}$$

$$F_{mq,z}(0,0,z) \propto -\Re\{\alpha_q^{(m)}\}\, k^3 H_0^2\, \sin(2kz). \tag{37}$$

Hence the multipole terms have the same spatial behavior but opposite signs between magnetic and electrical multipoles and alternate bewteen different orders as anticipated in α_{eff}.

III. OPTICAL SIMULATION

The structure of interest is shown in Fig. S2. The corresponding multipole polarizability and optical forces are numerically simulated using the RF module of the commercially available solver COMSOL Multiphysics. We employ a scattered field formulation, defining a meta-atom (M.A) composed of silicon discs with cross beams (n_{Si} = 3.48 + i 5.3 × 10^{-11}[3]) suspended in air ($n_0 = 1$), with periodic boundary conditions (PBC) applied in the x and y directions. Perfectly matched layers (PML) are implemented along $\pm z$ to minimize boundary reflections. A standing wave at normal incidence sets the background field. The simulation uses a mesh size of $\lambda/(20n)$, where λ is the wavelength of the standing wave illumination, and n is the refractive index of the respective material. The total field (scattered + background) is extracted from the simulation to compute the polarizabilities and optical forces (see Sec. III A). Multipole polarizabilities are obtained using the multipole decomposition method [4, 5], while optical forces are determined via the Maxwell stress tensor formulation [6].

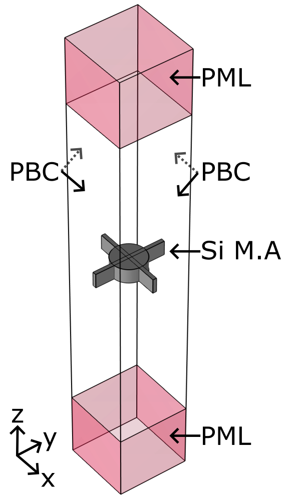

Figure S2. **Simulation settings** Unit cell of the meta-atom (Si M.A) structure, consisting of silicon discs with cross beams suspended in air. Periodic boundary conditions (PBC) are applied in the x and y directions, while perfectly matched layers (PML) are used along $\pm z$ to suppress reflections. A standing wave at normal incidence provides the background field.

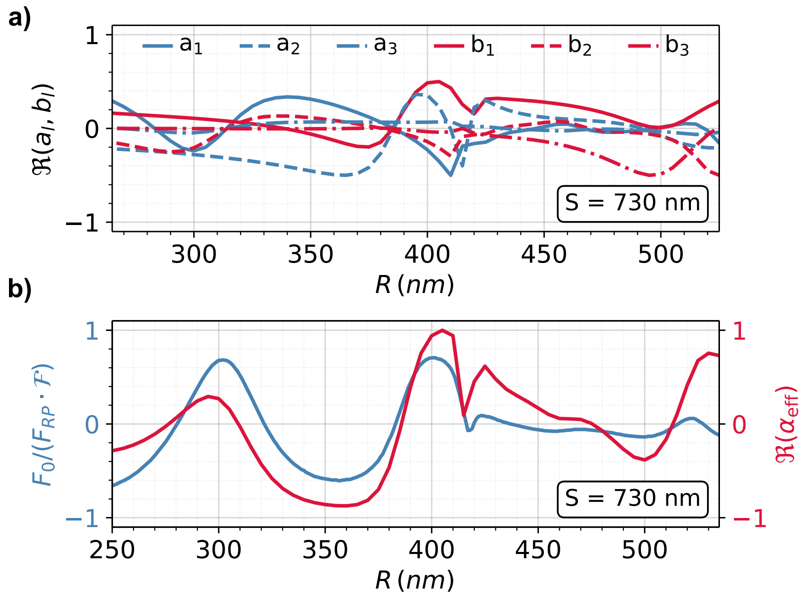

Figure S3. **Multipole polarizabilities and optical forces a)** Mie scattering coefficients $\Re(a_l, b_l)$ as a function of radius R and separation $S = 730\,\text{nm}$. **b)** Comparison between the effective polarizability $\Re(\alpha_{\text{eff}})$ (red solid line) and normalized optical forces $F_0/(F_{RP}\mathcal{F})$ (blue solid line) as a function of radius R and separation $S = 730\,\text{nm}$.

A. Mie resonances and polarisability

We simulate the effective polarizability of the membrane in COMSOL simulations and find it well approximated by the linear combination [7, 8]

$$\alpha_{\text{eff}} \approx [\alpha_d^{(m)} - \alpha_d^{(e)}] - [\alpha_q^{(m)} - \alpha_q^{(e)}] + [\alpha_o^{(m)} - \alpha_o^{(e)}]$$

where $\alpha_d^{(m)}$ and $\alpha_d^{(e)}$ correspond to the magnetic and electric dipole contributions, $\alpha_q^{(m)}$ and $\alpha_q^{(e)}$ to the magnetic and electric quadrupoles, and $\alpha_o^{(m)}$ and $\alpha_o^{(e)}$ to the magnetic and electric octupole. These are the dominant resonances considered in our analysis [7, 8]. Figure S3a illustrates the Mie scattering coefficients corresponding to individual multipole contributions to the effective polarizability as a function of the particle radius R, for a representative

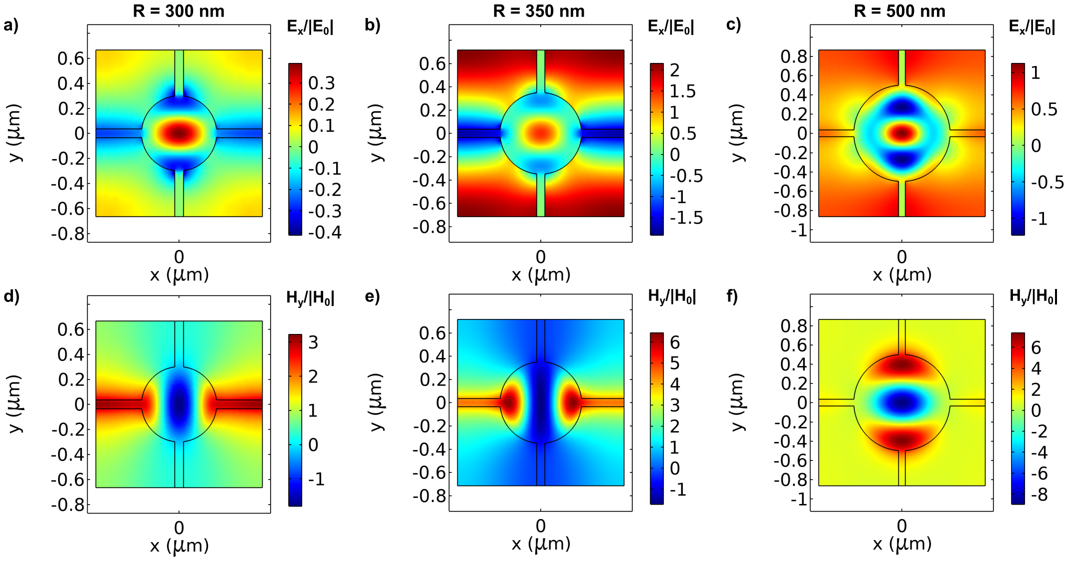

Figure S4. **Near-field electric and magnetic distributions.** Normalized electric (**a**, **b**, **c**) and magnetic (**d**, **e**, **f**) field profiles for disk radii of $R = 300$, 350, and 500 nm, respectively. The fields are normalized to the background standing-wave amplitude $|E_0|$ and $|H_0|$.

separation distance of $S = 730\,\text{nm}$. Here, a_l and b_l denote the electric and magnetic multipole coefficients, respectively, with $l = 1, 2, 3$ corresponding to dipolar, quadrupolar, and octopolar contributions. In Figure S3b we compare the real part of the simulated effective polarizability $\Re(\alpha_{\text{eff}})$ with the radiation pressure force F_0 normalized to the optical force experienced by a flat mirror F_{RP} and corrected by the filling factor $\mathcal{F}$. These figures highlight the direct competition between electric and magnetic resonances, which governs both the sign and magnitude of the effective polarizability and, consequently, the direction of the optical force. For instance, at $R \approx 300$ nm the dipolar term a_1 and b_1 dominates, yielding a positive force. The corresponding electrical and magnetic near-field profiles for $R = 300$ nm are shown in Fig. S4a and d, respectively, indicating a mix of electric and magnetic dipoles. While at larger radii, for example at $R = 350$ nm, the quadrupolar channel a_2 and b_2 overtakes, reversing the sign of $\Re(\alpha_{\text{eff}})$ and thus the optical force. The associated electrical and magnetic near-field profiles are shown in Fig. S4b and e, respectively. Additionally, at $R = 500$ nm, the magnetic octupole channel b_3 is dominating (see Fig. S4c and f for near-field mode profile) with negative polarizability and giving rise to negative force.

To further elucidate the origin of the force reversal, we analyze the far-field scattering patterns corresponding to the cases of positive and negative optical forces, as shown in Fig. S5. The magnitude of the electric field along the optical standing wave is displayed in Fig. S5a, revealing the expected sinusoidal intensity distribution and $z = 0$ corresponds to the positive intensity slope.
For the case of a positive optical force ($R = 300\,\text{nm}$), the scattered light is predominantly scattered backwards (Fig. S1b), which pushes the metastructure towards $z > 0$ (towards the intensity maxima), consistent with momentum conservation. In contrast, when the optical force becomes negative ($R = 350\,\text{nm}$), the scattering is mainly directed forward (Fig. S1c), thereby exerting a recoil force that drives the metastructure towards $z < 0$ (towards the intensity minima). This directional redistribution of scattered light provides a clear physical interpretation of how the interplay between electric and magnetic multipoles governs the sign of the optical force.

B. Polarization independence of optical force

The metasurface unit cell is designed to be laterally symmetric in the xy-plane. Owing to this symmetry, the structure exhibits an identical optical response for any polarization, resulting in an optical force along the z-direction that remains invariant in both magnitude and sign.
To confirm this behavior, Fig. S6 compares the normalized simulated z-component of the optical force, F_0, under x-polarized, y-polarized and right-handed circularly polarized (RHCP) illumination. As shown, F_0 is identical in both magnitude and direction for the three polarization states, verifying the polarization-insensitive nature of the normal optical force.

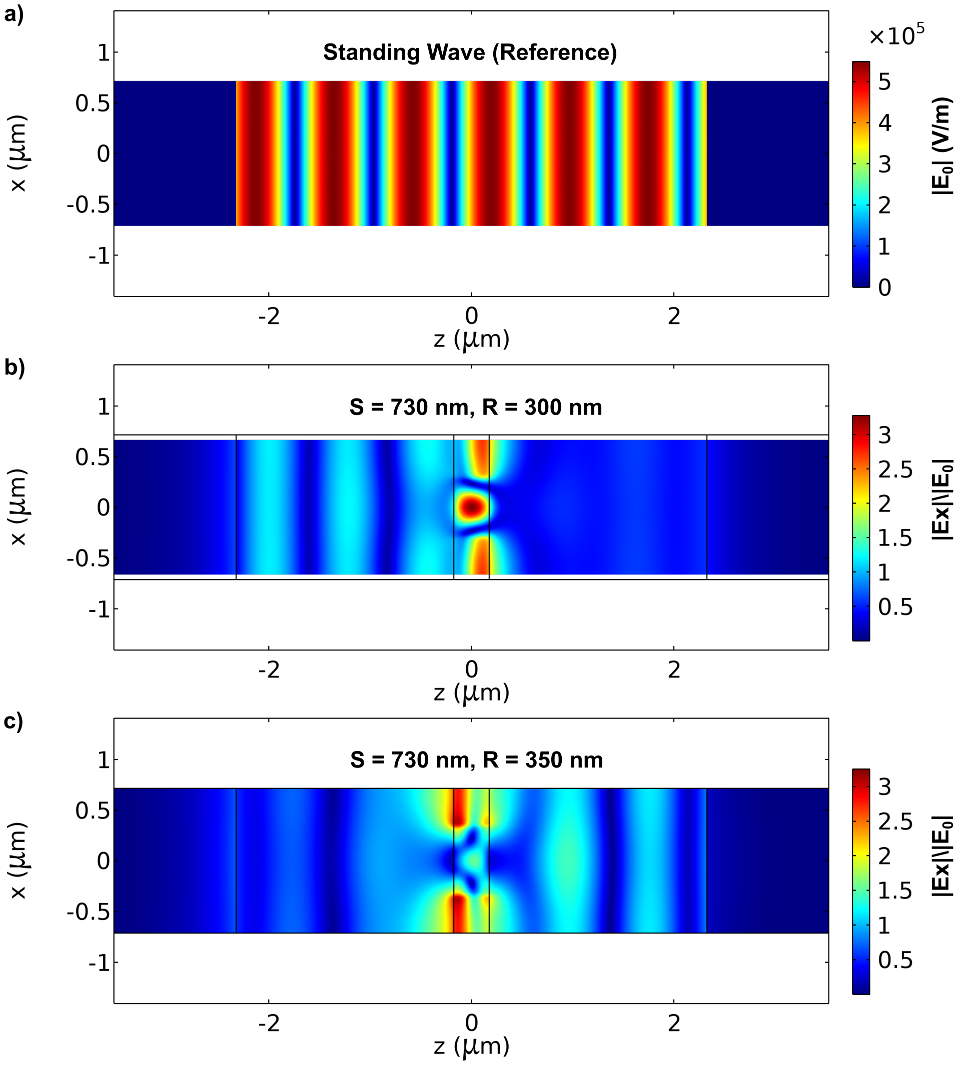

Figure S5. **Directional scattering of meta-atoms.** **a)** Electrical field $|E_0|$ of the optical standing wave with the positive intensity slope at $z = 0$. **b)** Positive force towards $z > 0$ for $R = 300$nm and $S = 730$nm. **c)** Negative force towards $z < 0$ for $R = 350$nm and $S = 730$nm. The central solid lines indicate the membrane boundaries and the outer solid lines perfect match layers.

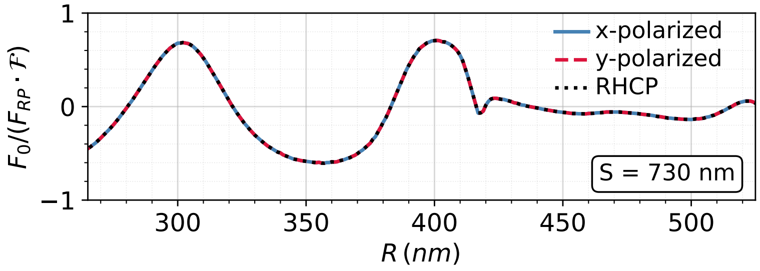

Figure S6. **Polarization independence of optical force**. The optical force F_0 for different radius R under linear polarization along x (solid blue), y (dashed red) and right-hand circular polarization RHCP (dotted black).

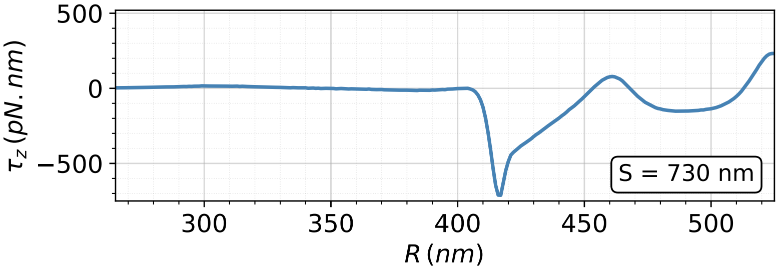

Figure S7. **Emerging torque under circular polarization**. Torque τ_z for different radius R under right-hand circular polarization (RHCP). The torque assumes positive and negative values.

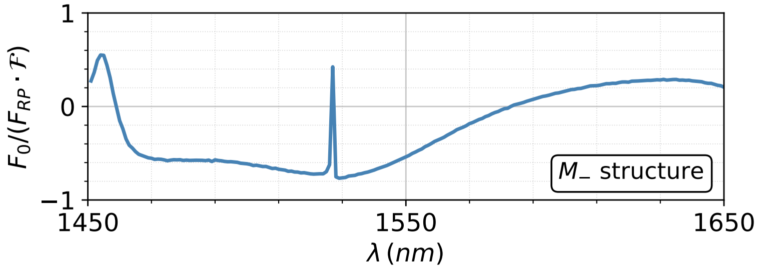

Figure S8. **Wavelength dependence of the optical force.** The normalized optical force $F_0/(F_{RP}\mathcal{F})$ as a function of the wavelength λ for the M_{-} structure under linearly polarized (x-polarized) illumination.

C. Optical torque under circular polarization

We note that circularly polarized light transfers spin angular momentum from the light to the metasurface. As a result, an optical torque component, τ_z, arises, as depicted in Fig. S7. This torque can be tuned from positive to negative values by adjusting the geometrical parameters.

D. Wavelength dependence of the optical force

To exploit the fact that Mie resonances are governed by the ratio between the wavelength and the structural dimensions, one can tune the illumination wavelength to access both positive and negative optical forces for a fixed metasurface design. Fig. S8 illustrates this principle by showing the normalized optical force $F_0/(F_{RP}\mathcal{F})$ as a function of wavelength for the representative M_{-} structure under x-polarized illumination. The wavelength sweep reveals a pronounced tunability of both the magnitude and the sign of the optical force, demonstrating simple spectral control between attractive and repulsive forces.

E. Optical force in the visible regime

To explore the general applicability of the negative force concept in high refractive index particles we extend our simulations to the visible range. As example, we simulate the optical force along the propagation axis at the common visible wavelength of $\lambda = 632$nm, while sweeping the disk radius R from 250 nm to 550 nm at fixed periodicity ($S = 730$ nm). The results are presented in Fig. S9. Since the lattice separation S is larger now than the wavelength λ, the meta-atoms behave more like isolated scatterers. Consequently, collective effects are relatively weak in this regime which results in reduced optical forces F_0 compared to larger wavelengths. Nevertheless, the direction of the optical force can still be controlled by tuning the geometrical parameters of the meta-atoms, confirming the principle of optical force reversal across a broad wavelength range.

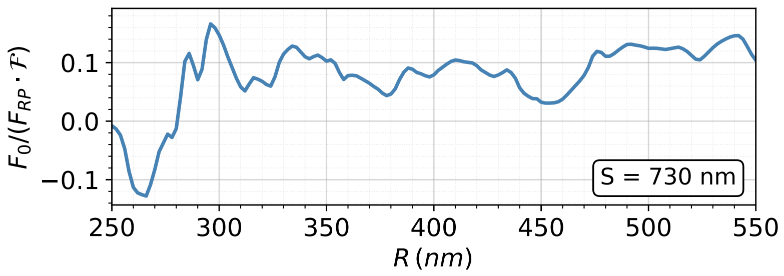

Figure S9. **Transversal optical force at visible wavelength:** F_0 versus radius R for separation $S = 730$nm at wavelength $\lambda = 633$nm.

IV. DRIVEN UNDERDAMPED HARMONIC OSCILLATOR

The equation of motion for a damped harmonic oscillator is:

$$\ddot{z}(t) + \Gamma\dot{z}(t) + \Omega_m^2 z(t) = \frac{F_0}{2m}\left(1 + \cos(\omega_{\mathrm{dr}}t)\right), \tag{38}$$

where Ω_m is the mechanical eigenfrequency, m is the mass, Γ is the damping coefficient, and the external driving force $F(t) = F_0/2(1 + \cos(\omega_{\mathrm{dr}}t))$ at driving frequency ω_{dr} and force amplitude F_0. The driving force $F(t)$ maintains a constant sign, which is set by F_0. The steady-state solution for $z(t)$ can be written as:

$$z(t) = \mathcal{A}(\omega)\cos(\omega t + \theta(\omega)),$$

where $\mathcal{A}(\omega)$ is the amplitude and $\theta(\omega)$ is the phase. Substituting this into the equation of motion, we obtain:

$$\mathcal{A}(\omega) = \frac{F_0}{2m\sqrt{(\Omega_m^2 - \omega^2)^2 + (\Gamma\omega)^2}}, \tag{39}$$

and the phase shift is given by:

$$\theta(\omega) = \tan^{-1}\left(\frac{\Gamma\omega}{\Omega_m^2 - \omega^2}\right). \tag{40}$$

Depending on the design parameters, the membrane acts as either a high-field (maximum intensity) or low-field seeker (minimum intensity), with the direction of its displacement determined by the sign of the intensity gradient at position z_0 along the standing wave. While the amplitude and phase response remain formally unchanged for both cases when comparing $z(t)$ and $F(t)$, low-field seekers move in the opposite direction (toward $z < z_0$) compared to high-field seekers. But since measurements are referenced to the driving signal controlling $I(t)$, this results in an apparent reversal of the external force and a phase shift in the observed motion relative to the modulation signal.

$$\Theta(\omega) = \theta(\omega) + \phi$$

with $\phi = 0$ or π depending on position and nature of the membrane. We refer to $\phi = 0$ ($\phi = \pi$) to positive (negative) force.

V. AMPLITUDE AND PHASE RESPONSE OF FLAT MIRROR MEMBRANE

In the main manuscript we normalize the amplitude response of the metasurfaces F_0 with the amplitude response of a flat mirror membrane F_{RP} (see Fig. S10a). The mirror amplitude and phase response to the optical drive at ω_{dr} is depicted in Fig. S10b and c, respectively. The flat mirror membrane without meta-atoms experiences the standard radiation pressure force.

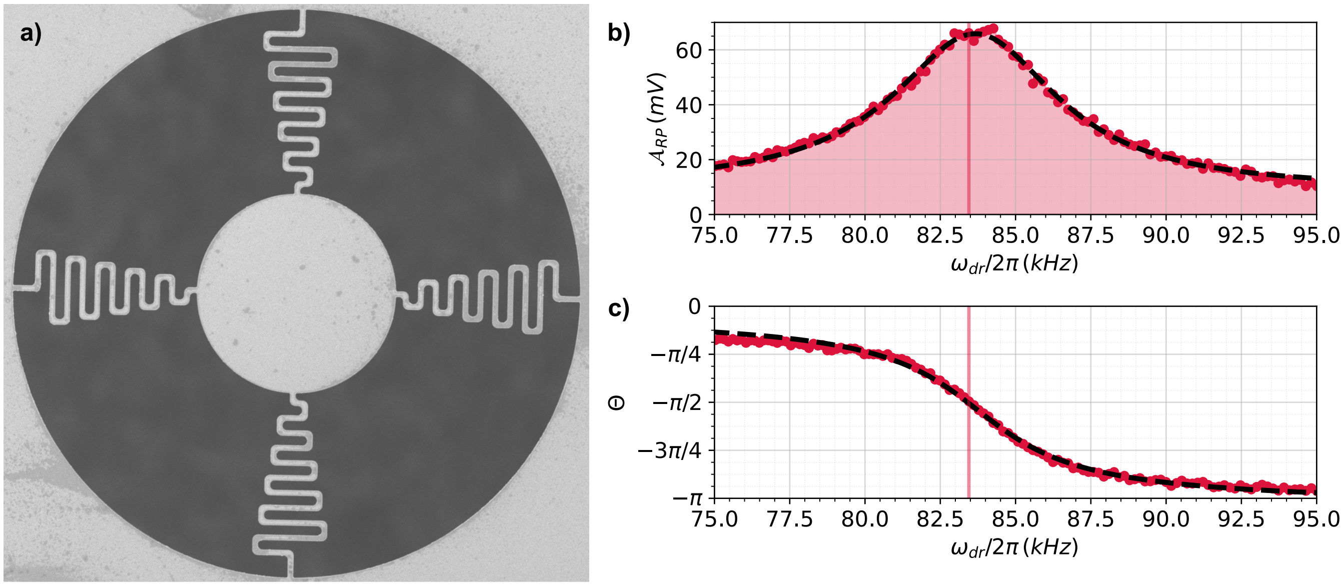

Figure S10. **Optically driven response of the flat membrane.** **a)** SEM micrograph of a flat membrane structure. **b)** Amplitude $\mathcal{A}_{RP}$ of the flat membrane as a function of driving frequency ω_{dr}. **c)** Phase Θ of displacement $z(t)$ of the flat membrane as a function of driving frequency ω_{dr}. The red vertical lines highlight the mechanical resonance frequency (Ω_m) and the black dashed lines represent a fit to Eqs. 39-40.

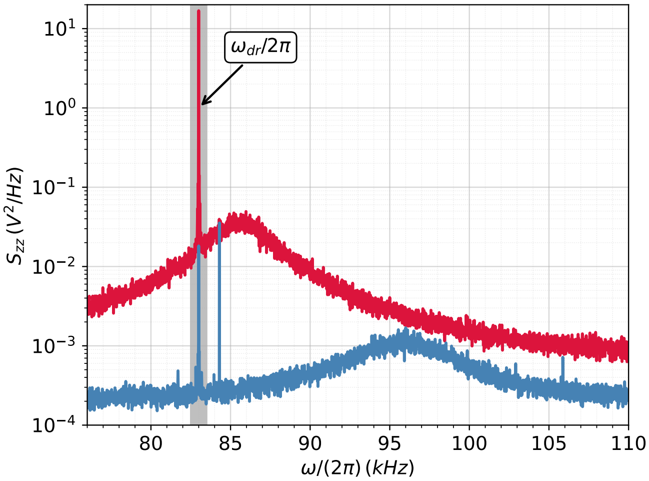

Figure S11. **Driven thermal motion of structured and flat membranes.** Measured power spectral density (S_{zz}) of a structured membrane with radius $R = 445\,\text{nm}$ and separation $S = 730\,\text{nm}$ (blue), compared to a flat membrane (red). Both membranes are driven at a fixed frequency $\omega_{dr}/2\pi = 83\,\text{kHz}$. The shaded gray region highlights the enhanced mechanical response at the driving frequency, demonstrating a significantly stronger signal compared to the thermally driven background.

VI. THERMALLY VERSUS OPTICALLY DRIVEN AMPLITUDE RESPONSE

In our model we neglect the thermal motion compared to the amplitude response under the external, optical drive. To demonstrate the validity of this claim, the power spectral density of a thermally driven mirror membrane and a structured membrane under the additional optical driving at $\omega_{\text{dr}}/2\pi = 83\,\text{kHz}$ has been measured. The chosen metasurface experiences the lowest value of the measured optical forces, giving a lower bound. Figure S11 shows the measured power spectral density S_{zz} for both a mirror membrane (red) and a structured membrane with radius $R = 445\,\text{nm}$ and separation $S = 730\,\text{nm}$ (blue). We find that the driven mechanical response (indicated by the gray shaded region) significantly exceeds the thermal background by nearly 2-3 orders of magnitude.

[1] Y. Jiang, J. Ng, and Z. Lin, Ab initio derivation of multipolar expansion of optical force, arXiv preprint arXiv:1512.04201 (2015).

[2] Note that, here we have neglected the toroidal dipole.

[3] J. Degallaix, R. Flaminio, D. Forest, M. Granata, C. Michel, L. Pinard, T. Bertrand, and G. Cagnoli, Bulk optical absorption of high resistivity silicon at 1550 nm, Opt. Lett. **38**, 2047 (2013).

[4] Y. F. Yu, A. Y. Zhu, R. Paniagua-Domínguez, Y. H. Fu, B. Luk'yanchuk, and A. I. Kuznetsov, High-transmission dielectric metasurface with 2π phase control at visible wavelengths, Laser Photonics Rev. **9**, 412 (2015).

[5] P. Grahn, A. Shevchenko, and M. Kaivola, Electromagnetic multipole theory for optical nanomaterials, New J. Phys. **14**, 093033 (2012).

[6] J. D. Jackson, *Classical electrodynamics* (John Wiley & Sons, 2021).

[7] J. Chen, J. Ng, Z. Lin, and C. T. Chan, Optical pulling force, Nat. Photonics **5**, 531 (2011).

[8] S. Lepeshov, N. Meyer, P. Maurer, O. Romero-Isart, and R. Quidant, Levitated optomechanics with meta-atoms, Phys. Rev. Lett. **130**, 233601 (2023).
